# Supplementary material for: The Evolution and Characterization of the RNA Interference Pathways in Lophotrochozoa
Source: Genome Biol Evol. 2024 May 7;16(5):evae098. doi: 10.1093/gbe/evae098 (PMC11114477; doi:10.1093/gbe/evae098)
Supplement: evae098_Supplementary_Data [file evae098_supplementary_data.zip › Supplementary_materials.zip!/selected_miRNAs/NoGe_selected_miRNAs/known_miRNA_families_pdf_files/NMRB01000470.1_Notospermus_geniculatus_strain_Ushimado_scaffold470,_whole_genome_shotgun_sequence_14115.pdf]

Provisional ID	:	NMRB01000470.1_Notospermus_geniculatus_strain_Ushimado_scaffold470,_whole_genome_shotgun_sequence_14115
Score total	:	29200.6
Score for star read(s)	:	3.9
Score for read counts	:	29192.2
Score for mfe	:	1.5
Score for randfold	:	0
Score for cons. seed	:	3
Total read count	:	57271
Mature read count	:	57255
Loop read count	:	0
Star read count	:	16

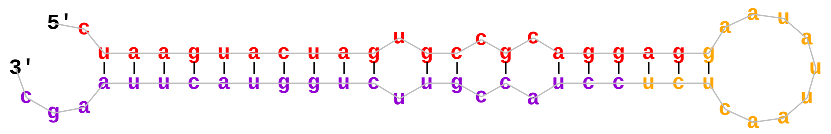

Genome coordinates: NMRB01000470.1 348687-348744

pre-miRNA sequence:
CUAAGUACUAGUGCCGCAGGAGCUAAGUACUAGUGCCGCAGGAGGAAUAUUAACUCUCCUACCGUUCUGGUACUUAAGC

Mature sequence: CUAAGUACUAGUGCCGCAGGAG

Aligned mature sequence of the *N. geniculatus* pre-miRNA with the top 5 hits from the BLAST search against the pre-miRNA mirGeneDB set:

```
                              **********************
Nge_Mir-252-P2                cuaaguacuagugccgcaggag-
Lan-Mir-252-P2_5p             cuaaguacuagugccgcaggagu
Pfl-Mir-252-P2_5p             cuaaguacuagugccgcaggagu
Sko-Mir-252-P2_5p             cuaaguacuagugccgcaggagu
Tca-Mir-252-P2_5p             cuaaguacuagugccgcaggag-
Cte-Mir-252-P2_5p             cuaaguacuagugccgcaggag-
```
